# Retrieval Practice Is Effective Regardless of Self-Reported *Need for Cognition* - Behavioral and Brain Imaging Evidence

**DOI:** 10.3389/fpsyg.2021.797395

**Published:** 2022-02-10

**Authors:** Carola Wiklund-Hörnqvist, Sara Stillesjö, Micael Andersson, Bert Jonsson, Lars Nyberg

**Affiliations:** ^1^Department of Psychology, Umeå University, Umeå, Sweden; ^2^Umeå Center for Functional Brain Imaging, Umeå, Sweden; ^3^Department of Applied Educational Science, Umeå University, Umeå, Sweden; ^4^Department of Integrative Medical Biology, Umeå University, Umeå, Sweden; ^5^Department of Radiation Sciences, Umeå University, Umeå, Sweden

**Keywords:** retrieval practice, testing effect, *need for cognition* (NFC), learning and memory, fMRI, classroom

## Abstract

There is an emerging consensus that retrieval practice is a powerful way to enhance long-term retention and to reduce achievement gaps in school settings. Less is known whether retrieval practice benefits performance in individuals with low intrinsic motivation to spend time and effort on a given task, as measured by self-reported *need for cognition* (NFC). Here, we examined retrieval practice in relation to individual differences in NFC by combining behavioral and functional magnetic resonance imaging (fMRI) data. Using a within-subject design, upper-secondary school students (*N* = 274) learned a language-based material (Swahili-Swedish word-pairs), with half of the items by means of retrieval practice with feedback and half by study only. One week later, the students were tested on the word-pairs either in the classroom (*n* = 204), or in a fMRI scanner (*n* = 70). In both settings, a retrieval practice effect was observed across different levels of NFC (high or low). Relatedly, comparable fMRI effects were seen in both NFC subgroups. Taken together, our findings provide behavioral and brain-imaging evidence that retrieval practice is effective also for individuals with lower levels of NFC, which is of direct relevance for educational practice.

## Introduction

Recent meta-analytic reviews have demonstrated that active learning methods reduce the achievement gap between academic success and failure (Freeman et al., 2014; Theobald et al., 2020). Similarly, key insights from neuroscience on learning and memory have shown that learning by actively engaging the brain has a direct effect on learning and memory retention (e.g., Mårtensson et al., 2012; Stillesjö et al., 2021). One form of active learning is retrieval practice, where the activity of including test sessions while acquiring new information has been shown to markedly boost long-term retention (i.e., commonly denoted as the testing-effect; e.g., see Roediger and Karpicke, 2006a,b; Roediger and Butler, 2011; Dunlosky et al., 2013; Agarwal et al., 2021; McDermott, 2021; for reviews).

The positive learning effects following retrieval practice have been demonstrated in: (1) young children ranging to older adults (e.g., Fazio and Marsh, 2019), (2) from easy to more complex materials (e.g., Karpicke and Aue, 2015; McDermott, 2021), (3) for both theoretical and practical course subjects (e.g., Dunlosky et al., 2013; Larsen et al., 2013), (4) for students with lower cognitive abilities (e.g., Brewer and Unsworth, 2012; Agarwal et al., 2017; Jonsson et al., 2020) as well as for those (5) with a diversity of learning disabilities (e.g., ADHD; Knouse et al., 2016, Downs syndrome; Starling et al., 2019, dyslexia/development language disorder; Leonard et al., 2019) and (6) to result in better learning outcome compared to other learning active methods [e.g., group discussions (Stenlund et al., 2017) and mind maps (Karpicke and Blunt, 2011)]. Based on the available evidence, it has been argued that retrieval practice is a learning method that is easy to apply and, as such, has high utility for educational practice across ages and course subjects (see also Dunlosky et al., 2013; Moreira et al., 2019, McDermott, 2021; for examples of reviews and meta-analyzes). In spite of this evidence, both students and teachers tend to overlook the beneficial effects of retrieval practice, and instead think of it as a method for evaluation (i.e., summative assessment) than for learning (i.e., formative assessment; McDermott, 2021).

Despite the well-established learning effects retrieval practice has on long term retention, (i.e., the testing effect), less is known about its effect related to individual variations in need for cognition (NFC; Cacioppo et al., 1996). NFC is a personality trait and is defined as “*differences among individuals in their tendency to engage in and enjoy thinking*” (Cacioppo and Petty, 1982, p. 116). High levels of NFC have a positive impact on performance (Weissgerber et al., 2018) and school grades (Grass et al., 2017; Luong et al., 2017; Strobel et al., 2019). Whereas some evidence exists for a positive link between NFC and cognitive ability (e.g., Fleischhauer et al., 2010; Hill et al., 2016), others have proposed that there is no such relationship (e.g., Gärtner et al., 2021). For example, Gärtner et al. (2021) suggest that NFC is a trait that is less characterized by cognitive abilities *per se*, instead they rather stress that the degree of NFC is related to the willingness to invest effort and self-control in the task at hand (see also Sandra and Otto, 2018). Related to NFC, prior behavioral studies have reported that students with lower NFC have a tendency to prefer learning strategies characterized by surface rather than deep learning (e.g., Evans et al., 2003; Sandra and Otto, 2018), or lack engagement in cognitively demanding learning activities (e.g., Gärtner et al., 2021). Moreover, Gonthier and Roulin (2020) further provide evidence that individuals with lower levels of working memory capacity and NFC are more inclined to use less effective learning strategies given the task at hand (see also Evans et al., 2003 for related findings). As such, one challenge within the educational field is to identify and examine whether specific learning methods can reduce the influence intrinsic motivation to spend low cognitive effort on a given task has, and in turn boost learning and retention in individuals with lower NFC. One possible learning method for this purpose could be retrieval practice.

Recently, non-invasive brain imaging methods such as functional magnetic resonance imaging (fMRI) has served as a complementary method to study *how* and *why* retrieval practice benefit long-term retention. For example, activity differences for retrieval practice, relative study, have been observed in a number of cortical (e.g., Keresztes et al., 2014; Jonsson et al., 2020; see van den Broek et al., 2016 for an overview); and subcortical brain regions (e.g., Wing et al., 2013; Liu et al., 2014; Jonker et al., 2018; Wiklund-Hörnqvist et al., 2020) typically associated with semantic processing and retrieval of well-consolidated memory representations (see e.g., Cabeza et al., 2008; Binder and Desai, 2011; Eichenbaum, 2017). For example, Karlsson Wirebring reported higher functional brain activity in the inferior frontal gyrus (IFG) 1 week after retrieval practice. Activity in the IFG is associated with the reinstatement of semantic memory representations stored elsewhere in the brain, including the parietal and temporal cortices (Martin and Chao, 2001; Binder et al., 2009). Repeated retrieval has also been linked to subcortical brain regions such as the hippocampus (Wing et al., 2013; Liu et al., 2014; Vestergren and Nyberg, 2014; Jonker et al., 2018; Wiklund-Hörnqvist et al., 2020). In addition, it was recently suggested that retrieval practice strengthens subsequent memory *via* a dual action of the hippocampus to support retrieval of detailed as well as generalized memory representations (Wiklund-Hörnqvist et al., 2020). The positive learning effects 1 week after retrieval practice was recently demonstrated to be accompanied by higher brain activity in fronto-parietal brain regions independent of cognitive proficiency (Jonsson et al., 2020). However, it still remains unknown if a similar pattern of brain activity following retrieval practice can be observed for individuals reporting different levels of NFC.

**FIGURE 1:**
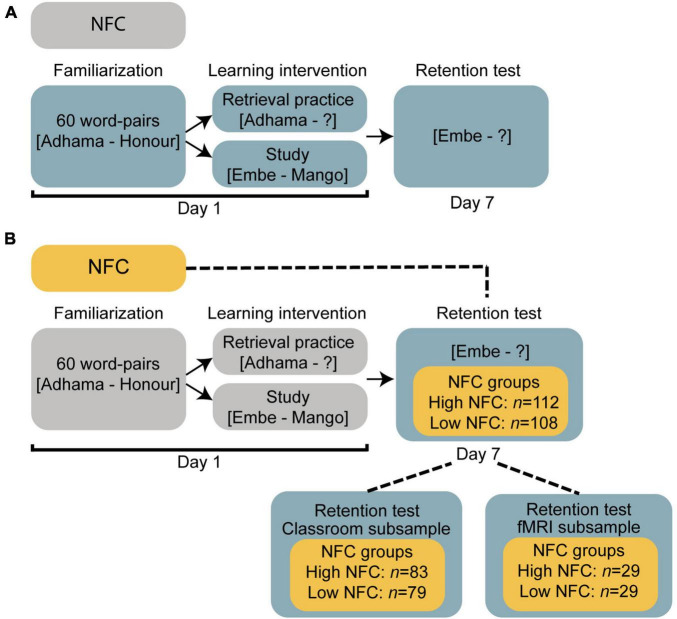
A schematic overview over the **(A)** overall study design and **(B)** related to the low and high NFC groups (yellow).

**FIGURE 2:**
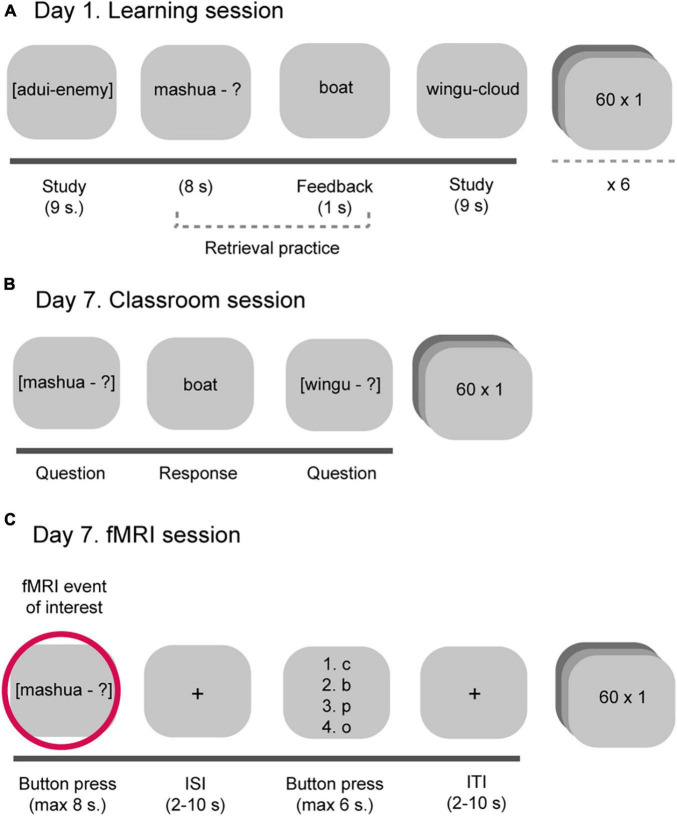
The experimental procedure related to **(A)** the learning intervention (day 1) and the 1 week retention test in the **(B)** classroom and the **(C)** MR scanner.

We here extend a previously published study (Jonsson et al., 2020) which focused on the retrieval practice effects related to cognitive ability and fMRI data. From the same data set, we here extracted a measure of NFC and examined individual differences in NFC in relation to the retrieval practice effects by combining behavioral and functional brain imaging data. Upper secondary school students (*N* = 274) participated in a learning intervention (study/retrieval practice) in the classroom. The to-be-learned material was foreign language vocabulary (60 Swahili-Swedish word-pairs). In the classroom, students learned half of the word-pairs by study, and the other half by retrieval practice and feedback (correct answer feedback). In both conditions, each word-pair was randomly presented six times, and interleaved between the two conditions. To examine the testing effect, learning was assessed by means of a cued recall test either in the classroom (*n* = 204) or by the use of fMRI (*n* = 70) 1 week after the learning intervention (see Figures 1, 2).

We have for this sample shown that brain activity is higher in several cortical and subcortical brain regions following retrieval practice (Jonsson et al., 2020; Wiklund-Hörnqvist et al., 2020). Several of the identified brain regions, such as the IFG and the hippocampus, have been implicated in retrieval of well-established semantic memories (Martin and Chao, 2001; Binder and Desai, 2011; Eichenbaum, 2017). One possibility is that individuals with high NFC will benefit more from retrieval practice, for example due to being more inclined to use semantic elaboration. If so, this would result in a significant fMRI main-effect of NFC group, for example reflecting higher activity in fronto-parietal brain regions and the hippocampus for high NFC individuals. An alternative possibility is that low NFC individuals will benefit more from retrieval practice, as the difference between a more passive (study) versus an active (retrieval practice) condition will be more marked for these individuals if retrieval practice “automatically” confers semantic elaboration. If so, this would yield a significant interaction effect between learning condition and NFC group, possibly in the IFG and hippocampus. Still another possibility is that retrieval practice will be equally effective regardless of level of NFC. Based on behavioral studies confirming the benefits of retrieval practice across a diversity of factors (see e.g., Roediger and Butler, 2011; Dunlosky et al., 2013; Fazio and Marsh, 2019; Moreira et al., 2019; Jonsson et al., 2020; Agarwal et al., 2021; McDermott, 2021; for overviews), we predicted that we would find support for the latter possibility, i.e., significant testing effects regardless of the level of NFC. If so, this could result in a significant fMRI main-effect (in favor for retrieval practice) with higher brain activity in IFG and hippocampus independent of NFC status. Alternatively, similar behavioral testing effects in individuals with high or low NFC levels could still map on to qualitative and/or quantitative differences in the recruited functional brain networks (i.e., behavioral equivalence does not always correspond to neural equivalence; Sohn et al., 2004). The combination of behavioral and fMRI data allowed us to assess the latter possible outcome.

## Materials and Methods

### Participants

Participants were 274 upper secondary school students (*M*_age_ = 17.51 years, *SD* = 0.74; *n* = 137 girls). All participants were native Swedish speakers, and none reported prior experience with the Swahili language. Prior to the data collection, written informed consent were obtained from the participants in accordance with the Helsinki declaration. For the fMRI subsample (*n* = 70; 54% girls), all participants were neurologically healthy, right-handed by self-report, had normal or corrected-to-normal vision. For participants (*n* = 10) who had not yet attained a legal age of majority (18 years.), written informed consent was obtained from the participant and both caregivers.

### Materials

#### Foreign Language Vocabulary

The to-be-learned material was 60 Swahili-Swedish word-pairs translated from Nelson and Dunlosky (1994) and previously used (e.g., Vestergren and Nyberg, 2014; Karlsson Wirebring et al., 2015; Wiklund-Hörnqvist et al., 2017, 2020).

#### Need for Cognition

Need for cognition was measured by the Mental Effort Tolerance Questionnaire (METQ; Dornic et al., 1991; Stenlund and Jonsson, 2017), which is a Swedish adaptation of the original NFC scale (Cacioppo and Petty, 1982). The METQ encompasses 30 items rated on a 5-point Likert scale (1 = strongly disagree, 3 = neutral, and 5 = strongly agree). Eighteen of the 30 items are reversed scored. The individual NFC score is calculated as the sum of all items, with higher scores as indicative for more NFC. In line with psychometric studies on the Swedish version of METQ, the internal consistency in this study was good, Chronbach’s α = 0.87 (see Stenlund and Jonsson, 2017 for psychometric evaluation of the Swedish version of METQ).

### Procedure

Need for cognition was collected 1–2 weeks prior to the learning intervention (see Figure 1). The learning intervention took place over two sessions (learning intervention and a retention test) separated by 1 week. The learning intervention was identical for all participants (see Figure 2A). The procedure for the 1 week retention test differed among participants such that the majority of the participants (classroom subsample, *n* = 204) performed the retention test in the classroom in front of their computer (see Figure 2B), but a subsample (fMRI subsample, *n* = 70) performed the 1 week retention test in the MR scanner (see Figure 2C).

#### Learning Intervention (Day 1)

The learning intervention was performed in the classroom (see Figure 2A). Each student sat in front of their own computer spaced apart from their classmates. First, to familiarize the students with the to-be-learned material, each complete Swahili-Swedish word-pair was presented one by one on the computer screen once. Next, half of the words were learned through study (Adhama – Honor), and the other half through retrieval practice (Bahasha - ?) followed by correct answer feedback (Envelope). Each word-pair was presented six consecutive times, and exposure time for each word was equal in both learning conditions (9 s). To prevent item and order effects, words were randomly interspersed related to learning condition (retrieval practice, study) and each student had a unique learning list.

#### One Week Retention Test (Day 7)

One week after the learning intervention, learning was assessed by means of a cued recall test either in the classroom (*n* = 204) or by the use of fMRI (*n* = 70). The only difference between the methodologies (classroom and fMRI) for the cued recall test was how students responded (see Figures 2B,C). In the classroom, all students were required to type in the corresponding Swedish counterpart using their laptop. In the scanner, students were instead asked to respond by a button press whether they *knew*, *believed they knew* or *did not know* the Swedish counterpart (see Figure 2C). Next, a jittered cross-hair appeared on the screen (ISI, 2–10 s). Students were then asked to select among four alternatives to indicate the second letter that corresponded to the second letter in the retrieved Swedish counterpart (right middle finger). The second letter cueing was used to single out correctly remembered words that were successfully retrieved. The position of the correct answer relative to the lures systematically varied to avoid item order effects (see Karlsson Wirebring et al., 2015; Wiklund-Hörnqvist et al., 2017, 2020; Jonsson et al., 2020; Stillesjö et al., 2021). Next followed a jittered crosshair (ITI, 2–10 s) before the presentation of the next cue appeared on the screen. The fMRI session lasted for ∼ 45 min, and ended with structural images.

### Statistical Analyzes Related to Need for Cognition

One of the purposes with the study was to delineate the association between NFC and performance 1 week after the learning intervention, and a second purpose was to use fMRI data to further complement behavioral data related to the first purpose. As such, individual scores on NFC were analyzed both at the individual (i.e., continuous variable) and split into subgroups based on NFC levels (i.e., high NFC group, low NFC group).

First, independent of learning condition, students (*N* = 274) performance 1 week after the learning intervention was correlated with the individual NFC score. Next, to further evaluate whether individual levels of NFC influences the testing effect, students were divided into high and low NFC groups. The high NFC group (*n* = 112) was defined as the 40% individuals with the highest NFC scores (*M* = 116.13, *SD* = 7.73; range NFC_score_ = 106–138). The low NFC group (*n* = 108) was defined as the 40% individuals with the lowest NFC scores (*M* = 87.43, *SD* = 8.75; range NFC_score_ = 58–97; see Figure 1B). The motivation to divide the sample into 40% high and 40% low NFC individuals, and to exclude 20% in the middle, was related to us wanting to separate the groups of interest. We therefore avoided using under/above the median to define “low” or “high” NFC individuals (but see Supplementary Figures 1A,B for an illustration including all participants).

Next, as the fMRI subsample (*n* = 70) already was included in the high and low NFC groups related to the behavioral analyzes (see Table 1 for descriptive statistics and Figure 1B), we re-run the ANOVA on the fMRI data.

**TABLE 1:** Descriptive statistics related to the testing effect, NFC scores for the different subsamples.

	NFC score		RP	S	TE
Sample	*M*	SD	*M* (SD)	*M* (SD)	*p*
Overall (*N* = 274)	101.99	14.87	0.46 (0.27)	0.30 (0.24)	<0.001
***Subsample***					
Classroom (*n* = 204)	101.82	15.22	0.48 (0.28)	0.32 (0.25)	<0.001
fMRI (*n* = 70)	102.46	13.90	0.41 (0.23)	0.25 (0.20)	<0.001

*NFC, Need for cognition; RP, Retrieval practice; S, Study; TE, testing effect.*

### Image Acquisition

Images were acquired on a 3.0 T whole-body scanner (MR 750, GE Medical Systems) equipped with a head coil. T2* weighted images were collected with a single-shot GE-EPI sequence for BOLD imaging. The parameters used for the data collection were: echo time, 30 ms; repetition time, 2,000 ms; flip angle, 90°; FOV, 248 × 248 mm; acquisition matrix 96 × 96 (reconstructed to 128 × 128 and hence 1.95 mm resolution); and slice thickness, 3.4 mm (37 slices acquired). Ten dummy scans were collected to allow equilibrium of the fMRI signal, and discarded before the start of the data collection. T1-weighted structural images were obtained for each participant. Cushions within the head coil were used to minimize head movements during scanning, and headphones and earplugs were used to reduce scanner noise. All stimuli were presented to the participants through a mirror attached to the head coil, and run from a PC through E-prime version 2.0 (Psychology Software Tools). Participants’ responses were collected with a four-key button keypad (Lumitouch fMRI optical response keypads, Photon Control).

Functional data were preprocessed in SPM 12 and run through an in-house program (DataZ). Preprocessing of all images included: Correction for slice-timing, and head movements were corrected with realign and unwarp. Segmentation was done for all T1-images, and a group specific mean template and individual flow fields were created with the DARTEL algorithm (Ashburner, 2007). The DARTEL template and flow fields were used to normalize the images to MNI space (2 mm), and the images were smoothed (8 mm FWHM Gaussian filter kernel).

### Functional Magnetic Resonance Imaging Data Analysis

A 2 (learning condition: Retrieval practice vs. Study) × 2 (NFC group: High NFC vs. low NFC) ANOVA was set up to examine patterns of brain activity change during retrieval practice in relation to self-reported NFC.

At the first level, for each student, individual general linear models were estimated. The model included separate regressors of interest (items learned through retrieval practice, items learned through study), and the six movement parameters were included as covariates of no interest. All regressors except the movement parameters were convolved with a hemodynamic response function. The design was event-related, and the duration was set to zero. Two *t*-contrast images were defined to evaluate brain activity specifically related to retrieval practice and study.

Second, to test for an interaction effect between learning conditions (retrieval practice, study) and NFC groups, a whole-brain 2 (retrieval practice, study) × 2 (high NFC, low NFC) ANOVA was performed. All students’ individual *t*-contrasts related to retrieval practice and study defined at the first level were inserted in the ANOVA. Peak activity related to retrieval practice and study in selected brain regions were plotted. The statistical threshold was set to *p* < 0.05 (FWE corrected), and *k* > 10. The ANOVA was also evaluated at a more liberal threshold *p* < 0.0001 (uncorrected) at the voxel level, and *k* > 10 at the cluster level.

## Results

### Behavioral Results

A paired *t*-test confirmed a significant testing effect [*t*(273) = 11.97, *p* < 0.001] meaning that performance was higher following retrieval practice compared to after study (see Table 1). Despite the significant testing effect, and independent of learning condition, individual variation in NFC was positively associated with long-term retention (study, *r* = 0.24, *p* < 0.001, retrieval practice, *r* = 0.25, *p* < 0.001).

**FIGURE 3:**
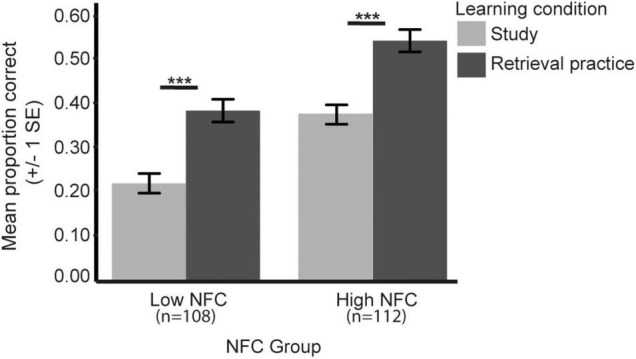
The behavioral testing effects related to the NFC groups. Error bars denote ± 1 SEM. ***p < 0.001.

To further evaluate whether different levels of NFC influences the testing effect, a mixed model ANOVA with learning condition (retrieval practice/study) as within-subject factor and NFC group (high/low) as between-subject factor was performed. Results revealed significant main effects of learning condition [*F*(1, 218) = 118.50, *p* < 0.001] and NFC group [*F*(1, 218) = 27.65, *p* < 0.001], but no significant interaction between NFC group and learning condition (*p* = 0.99; Figure 3). This means that independent of NFC group, significant testing effects were again confirmed, but also that the high NFC group (*n* = 112) displayed a higher performance level compared to the low NFC group (*n* = 108). As can be seen in Figure 3, the magnitude of the testing effect ([performance retrieval practice – performance study]) was identical for both NFC groups (High NFC: *M*_TE_ = 0.17, SE = 0.02; Low NFC: *M*_TE_ = 0.17, SE = 0.02), but the relative gain ([performance retrieval practice/performance study]) after retrieval practice was larger in the low NFC group (1.75) than in the high NFC group (1.44). Running the same analysis for the classroom (low NFC: *n* = 79; high NFC: *n* = 83) and the fMRI subsample (low NFC: *n* = 29; high NFC: *n* = 29) respectively, revealed comparable significant testing effects (*p’s* < 0.001, see Supplementary Figure 2). To further control whether the lack of the learning condition × NFC group interaction was plausible, a Bayesian mixed model ANOVA was performed on the whole sample. The analysis revealed a Bayes factor (BF_10_) of 0.14, providing weak support for a possible significant interaction effect (Lakens et al., 2020).

In sum, the behavioral analyzes showed that retrieval practice results in better long-term retention relative study independent of NFC, and that higher levels of NFC are related to higher performance independent of learning condition.

### Imaging Results

**FIGURE 4:**
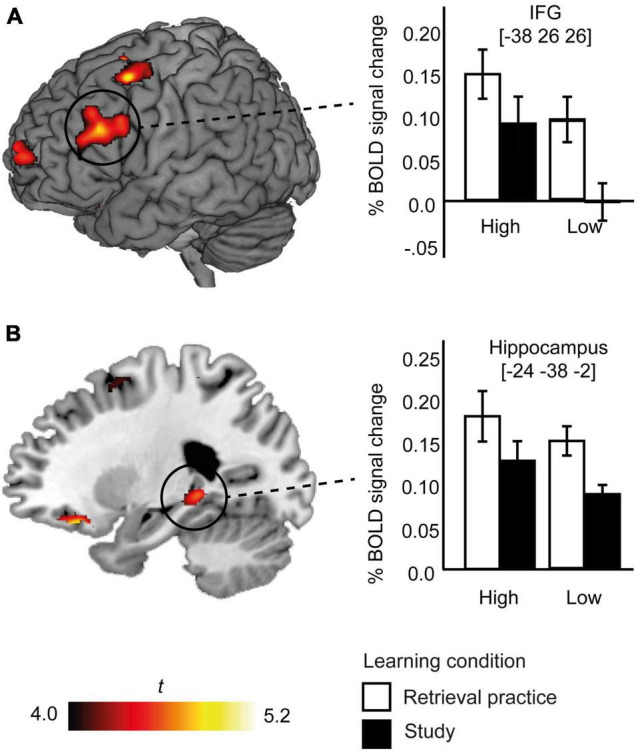
Brain activation related to the two learning condition (retrieval practice and study) × two NFC group (high NFC and low NFC) ANOVA. Brain activity more engaged after retrieval practice > study is illustrated in selected brain regions (**A**, the left IFG [−38 26 26] and **B**, the left hippocampus [−24 −38 −2]). BOLD activity for the retrieval practice and study conditions is plotted separately for the NFC groups (high and low). White bars represent the retrieval practice condition. Black bars represent the study condition. Error bars denote ± 1 SEM.

As expected, independent of performance (see Supplementary Figure 3), and in line with our previous analyzes of partly the same dataset (Jonsson et al., 2020), there was a significant main effect of condition, such that cued recall of items initially acquired by means of retrieval practice versus study engaged several left-lateralized cortical and subcortical brain regions (Figures 4A,B). There was no significant main effect of NFC group, but at the more lenient statistical threshold a main effect of NFC was observed in left precentral gyrus, (see Supplementary Figure 4).

The main focus of the imaging analysis was to evaluate if there was an interaction between initial learning condition (retrieval practice, study) and level of NFC. No significant interaction effect was found at the FWE corrected level, and not even at the more lenient statistical threshold (*p* < 0.0001, *k* > 10). Thus, differences in brain activity 1 week after acquisition by means of retrieval practice or study were independent of level of NFC. This outcome is illustrated for two regions from the main effect; the left IFG and hippocampus (Figures 4A,B). As can be seen, the difference in fMRI activity during cued recall of information acquired by retrieval practice versus study was of a comparable magnitude in the high and low NFC groups. To further control for possible interactions in the IFG and hippocampus, *post hoc* analyzes were performed. Beta values for the IFG and hippocampus from the main effect of condition were extracted for each participant and inserted in a 2 (retrieval practice, study) × 2 (low NFC, high NFC) ANOVA. No interactions were detected (left IFG: *p* = 0.25; left hippocampus = 0.73, respectively).

## Discussion

Using a within-subject design, we here combined behavioral methods with brain imaging to investigate the retrieval practice effects in relation to NFC among upper-secondary school students. A significant behavioral testing effect was confirmed for the whole sample, and dividing participants into low and high NFC groups revealed identical TEs in both groups, suggesting that retrieval practice seems to protect against lower levels of NFC. Imaging data further validated the behavioral observations, such that the difference in fMRI activity during cued recall of information acquired by retrieval practice versus study was of a comparable magnitude in the high and low NFC groups.

Our results clearly show that while NFC positively influences performance in general, it is unrelated to the magnitude of the testing effect. Evidence exists showing that retrieval practice, as a learning method, protects against acute stress (Smith et al., 2016; Pastötter et al., 2020), reduces test anxiety (i.e., examinations, see e.g., Agarwal et al., 2014; Szőllősi et al., 2017), reduces mind wandering (Szpunar et al., 2013) and has shown to be unrelated to different levels of cognitive abilities (Brewer and Unsworth, 2012; Agarwal et al., 2017; Bertilsson et al., 2020; Jonsson et al., 2020). Both NFC and the testing effect has each been studied extensively for their potential in memory and learning (e.g., Evans et al., 2003; Dunlosky et al., 2013; Sandra and Otto, 2018; Moreira et al., 2019; Strobel et al., 2019; Gonthier and Roulin, 2020; McDermott, 2021), but less is known of the association between the two. We here demonstrate that retrieval practice can boost learning and retention in individuals with lower NFC, possibly by enforcing active and deeper learning. Speculatively, retrieval practice as a learning method might prevent surface learning by requiring the learner to actively engage in the task at hand. As such, the learning method in itself might compensate for the lack of motivation and willingness to invest cognitive effort in a given task (Gärtner et al., 2021).

The present brain imaging results further extend the behavioral findings by showing that how the brain activates 1 week after learning with retrieval practice is comparable between different levels of NFC in upper-secondary school students. The lack of an interaction effect, as indicated by similar pattern of brain activity between NFC groups, further supports the finding that retrieval practice had an equal effect on the brain regardless of NFC. In addition, the significant main effect of retrieval practice (relative study) was evident in the left IFG and the left hippocampus. Such findings might support the idea that retrieval practice in itself prevents surface learning (Craik and Lockhart, 1972) as it requires the learner to actively engage in the task at hand, and more efficiently allocates the attention to stored memory representations regardless of NFC.

Both IFG and the hippocampus are well-established as brain regions implicated in learning and memory (e.g., Eichenbaum, 2017), and particularly in the retrieval of well-consolidated semantic memory representations (Martin and Chao, 2001; Salami et al., 2010, Binder and Desai, 2011). For example, a key role for the IFG in learning and memory is related to allocation of cognitive control for successful retrieval of memory representations stored elsewhere (Salami et al., 2010). In our prior fMRI studies we found support for that retrieval practice, as measured across three consecutive tests with (Wiklund-Hörnqvist et al., 2017) or without feedback (Karlsson Wirebring et al., 2015), reduces demands on left prefrontal brain regions implicated in cognitive control functions. Retrieval practice has also been found to increase hippocampal activity related to detailed and generalized memory representations 1 week after learning (Wiklund-Hörnqvist et al., 2020).

Taken together, our findings align with the positive learning effects retrieval practice has shown to have for students with lower cognitive abilities [e.g., working memory capacity (Agarwal et al., 2017) cognitive proficiency (Jonsson et al., 2020); general fluid intelligence (Brewer and Unsworth, 2012)], maintain executive control and prevents mind wandering during lectures (Szpunar et al., 2013). Those findings echo well with our brain imaging results related to NFC, which entails that brain regions in the IFG and hippocampus seems to be equally engaged for low and high NFC groups following retrieval practice. With that in mind, the general effectiveness of retrieval practice likely triggers neurocognitive mechanisms involved in enabling access to stored memory representations to a higher degree compared to study (Antony et al., 2017), regardless of NFC. Thus, combining behavioral data with brain imaging provide a unique window into the learning brain not possible to detect by behavioral data alone.

## Conclusion

In conclusion, we here provide behavioral and neurocognitive evidence that retrieval practice is effective for learning in the classroom regardless of levels of NFC. These results are promising for the educational field as they clearly demonstrate that learning by retrieval practice can limit the influence the willingness to invest cognitive effort has on performance, by boosting learning and retention in lower as well as high NFC individuals.

## Data Availability Statement

The raw data supporting the conclusions of this article will be made available by the authors, without undue reservation.

## Ethics Statement

The studies involving human participants were reviewed and approved by the Regional Ethical Review board, Umeå, Sweden. Written informed consent to participate in this study was provided by the participants’ legal guardian/next of kin.

## Author Contributions

CW-H and SS wrote the first draft of the manuscript. LN, BJ, and CW-H designed the research. CW-H performed the research. CW-H, SS, and MA analyzed the data. CW-H, SS, MA, BJ, and LN wrote sections of the manuscript. All authors contributed to the article and approved the submitted version.

## Conflict of Interest

The authors declare that the research was conducted in the absence of any commercial or financial relationships that could be construed as a potential conflict of interest.

## Publisher’s Note

All claims expressed in this article are solely those of the authors and do not necessarily represent those of their affiliated organizations, or those of the publisher, the editors and the reviewers. Any product that may be evaluated in this article, or claim that may be made by its manufacturer, is not guaranteed or endorsed by the publisher.

## Supplementary Material

Supplementary Figure 1 **(A)** The behavioral testing effect related to NFC group (high NFC and low NFC) reported in the article (i.e., Figure 3) along with an illustration of performance for the excluded intermediate NFC group (i.e., the 20% in the middle; patterned bars). Error bars denote ± 1 SEM., and **(B)** a plot for the association of each individual’s testing effect with their NFC score.

Supplementary Figure 2 The behavioral testing effect related to NFC group (high NFC and low NFC) and the different subsamples (classroom, fMRI). Error bars denote ± 1 SEM. ^***^*p* < 0.001.

Supplementary Figure 3 The overlap (yellow) in functional brain activity from the 2 × 2 ANOVA (main effect retrieval practice > study; red) and the group *t*-test controlling for performance differences (retrieval practice > study; green). For illustrative purposes, the effects are shown at a more lenient statistical threshold (uncorrected *p* < 0.0001 at the voxel level, and *k* > 10 at the cluster level).

Supplementary Figure 4 The main-effect of NFC group in the precentral gyrus [−48 4 32] at a more lenient statistical threshold (uncorrected *p* < 0.0001 at the voxel level, and *k* > 10 at the cluster level). Blue bars represent the retrieval practice condition. Red bars represent the study condition. Error bars denote ± 1 SEM.
