# Supplementary figures and images for: Retrieval Practice Is Effective Regardless of Self-Reported Need for Cognition - Behavioral and Brain Imaging Evidence
Source: Front Psychol. 2022 Feb 10;12:797395. doi: 10.3389/fpsyg.2021.797395 (PMC8866974; doi:10.3389/fpsyg.2021.797395)

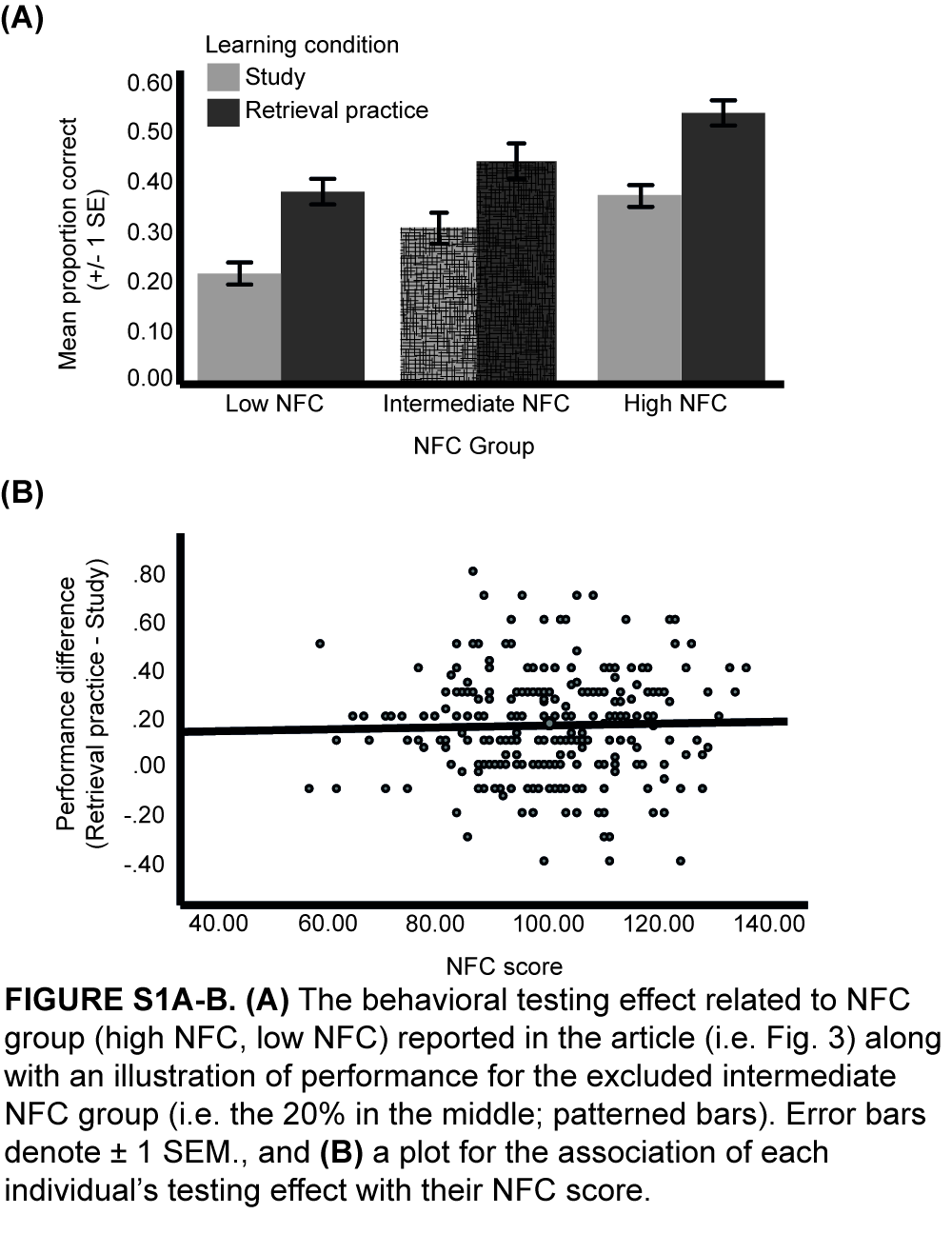

Supplement: Supplementary Figure 1 — (A) The behavioral testing effect related to NFC group (high NFC and low NFC) reported in the article (i.e., Figure 3) along with an illustration of performance for the excluded intermediate NFC group (i.e., the 20% in the middle; patterned bars). Error bars denote ± 1 SEM., and (B) a plot for the association of each individual’s testing effect with their NFC score. [file Image_1.tif]

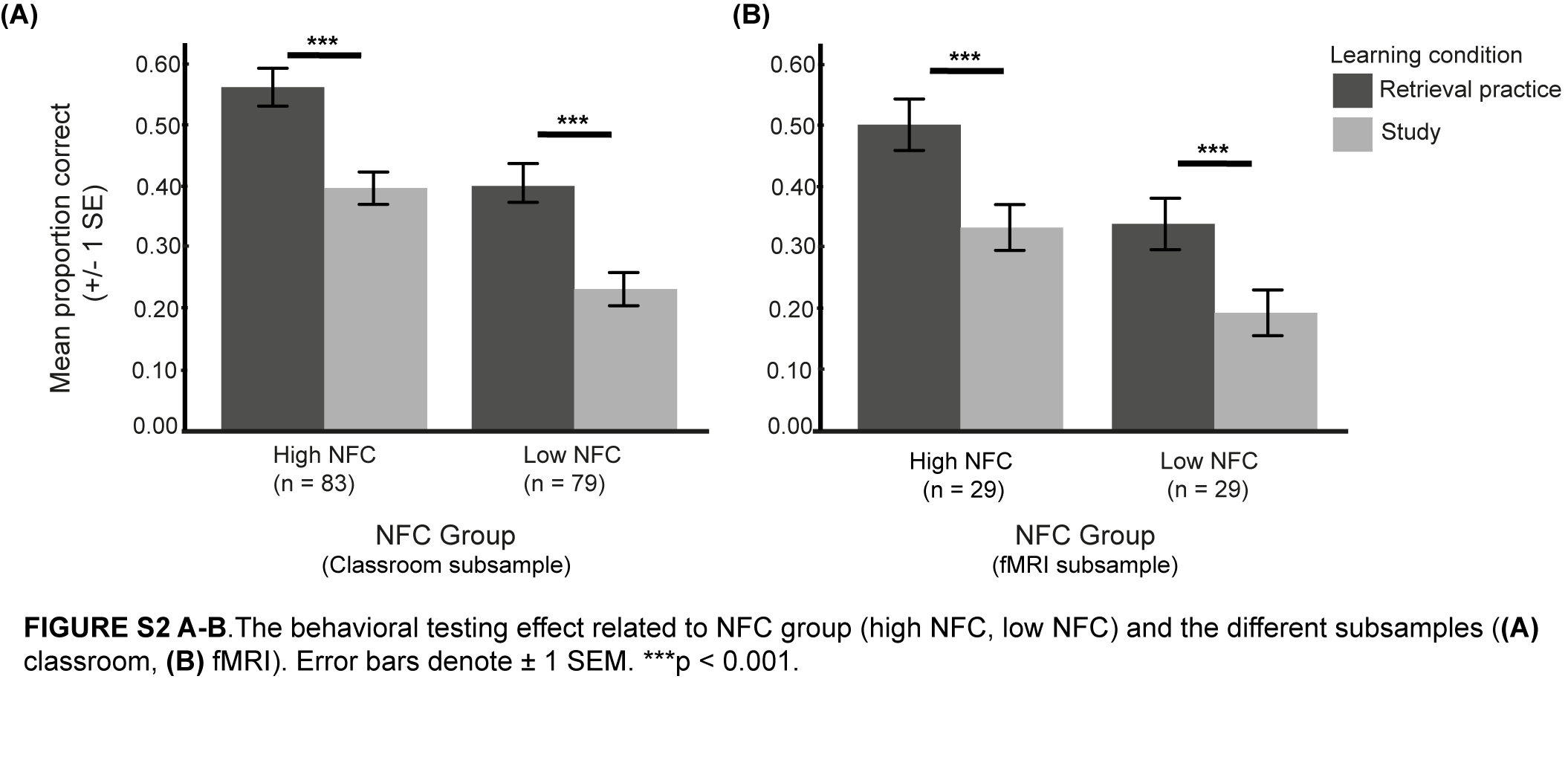

Supplement: Supplementary Figure 2 — The behavioral testing effect related to NFC group (high NFC and low NFC) and the different subsamples (classroom, fMRI). Error bars denote ± 1 SEM. ***p < 0.001. [file Image_2.tif]

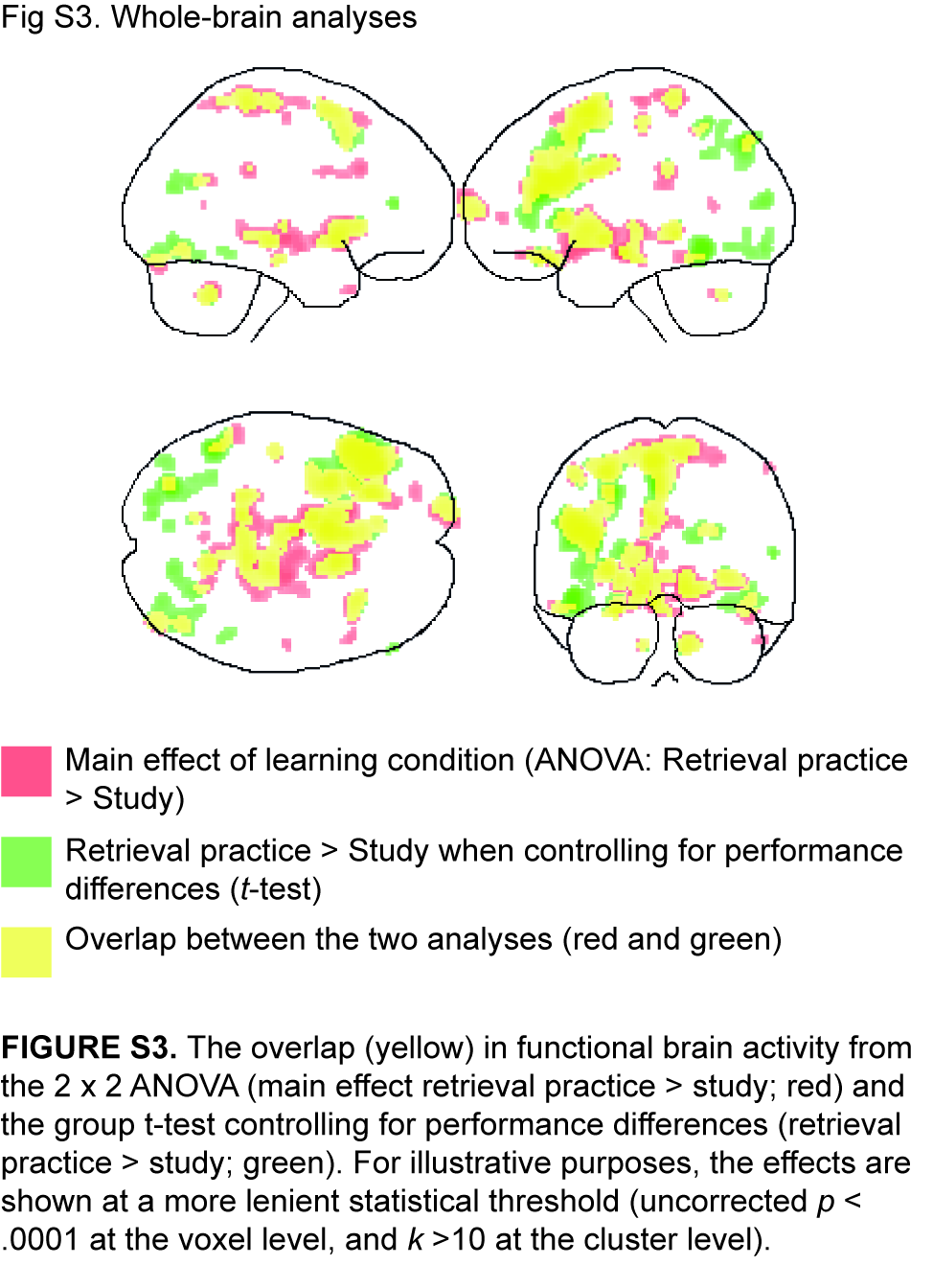

Supplement: Supplementary Figure 3 — The overlap (yellow) in functional brain activity from the 2 × 2 ANOVA (main effect retrieval practice > study; red) and the group t-test controlling for performance differences (retrieval practice > study; green). For illustrative purposes, the effects are shown at a more lenient statistical threshold (uncorrected p < 0.0001 at the voxel level, and k > 10 at the cluster level). [file Image_3.tif]

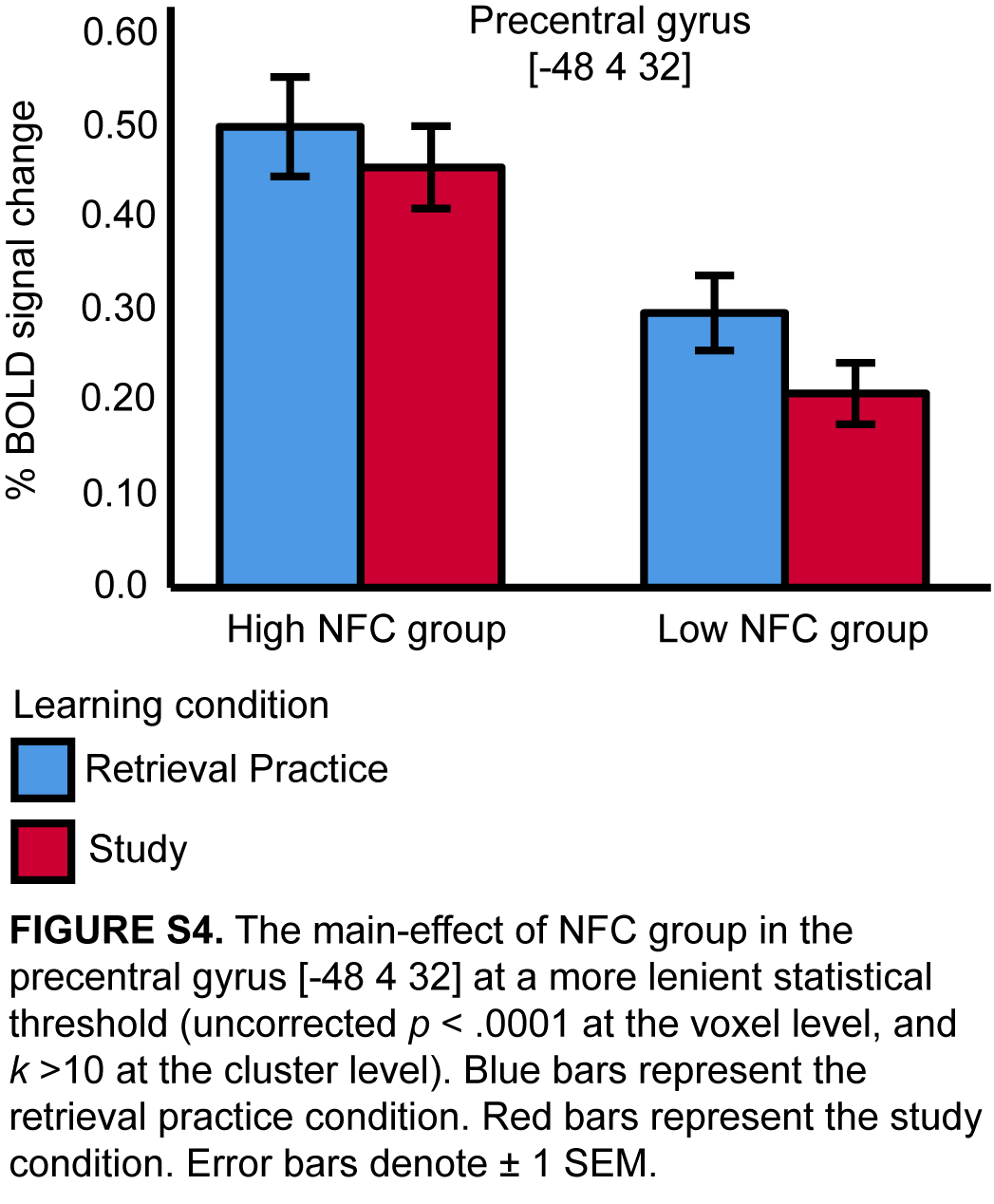

Supplement: Supplementary Figure 4 — The main-effect of NFC group in the precentral gyrus [−48 4 32] at a more lenient statistical threshold (uncorrected p < 0.0001 at the voxel level, and k > 10 at the cluster level). Blue bars represent the retrieval practice condition. Red bars represent the study condition. Error bars denote ± 1 SEM. [file Image_4.tif]
